# The Effect of Kinesio Taping on Handgrip and Active Range of Motion of Hand in Children with Cerebral Palsy

**Published:** 2018

**Authors:** Azadeh GHAHERI, Saman MAROUFIZADEH

**Affiliations:** 1 Department of Epidemiology and Reproductive Health, Reproductive Epidemiology Research Center, Royan Institute for Reproductive Biomedicine, ACECR, Tehran, Iran

**Dear Editor-in-Chief,**

With interest, we read the paper by Rasti et al. which aimed to investigate the effect of Kinesio taping on handgrip and active range of motion of hand in children with cerebral palsy (1). Here, we would like to emphasize only some important statistical and methodological points concerning the paper as follows:

Since this study was considered as an interventional trial on human, it should have been identified as a randomized clinical trial in title; describing the trial design details following the consort checklist (e.g. the method used for generating the random allocation sequence, details on type of randomization such as blocking, blinding, etc.) also seems absolutely necessary.

Because gender distribution in the two groups (male ratio, trail group: 33.3% vs. control group: 66.7%) was unbalanced, the applied allocation method was not suitable for this study. In such situations, the blocked randomization method is frequently used. Appropriate random assignment is critical in experimental designs because all factors beyond experimental assignments are equivalent and clarify the explanation of any observed difference between experimental groups. In this regard, if the blocked randomization method had been used the sex and age distributions would not have been asymmetric in the two groups (2).

Using the random allocation could avoid the difference between the trial and control groups on outcomes' pretest values. Regarding this difference between the two groups on pretest values of dependent variables, although not statistically significant, the authors should use the analysis of covariance (ANCOVA) to adjust the differences (3). 

In that study, *t*-test has been used to compare some demographic and clinical variables (such as age, sex, affected hand, cp type, etc.) between the two groups; however, this test could not be applied to compare categorical variables like sex. Descriptive statistics and tables must be also reported and designed in a standard format; for quantitative variables, the mean should be always reported along with a measure of variability (standard deviation or standard error) and for qualitative ones, frequency should be reported along with percent.

## Author’s contribution

Azadeh Ghaheri: Conception of the study, Drafting the article, Data interpretation.

Saman Maroufizadeh: Conception of the study, Drafting the article, Data interpretation.

Both authors approved the final version of the manuscript for publication**.**

## Conflict of interest

The authors declare that there is no conflict of interest.

## References

[1] Dalvand H (2017). The effect of Kinesio Taping on hand grip and active range of motion of hand in children with Cerebral Palsy. Iran J Child Neurol.

[2] Pocock SJ (2013). Clinical trials: a practical approach.

[3] Neter J, Kutner MH, Nachtsheim CJ, Wasserman W (1996). Applied linear statistical models.

